# Clinical and Pathological Reports of Cases of Insanity

**Published:** 1884-02

**Authors:** S. V. Clevenger

**Affiliations:** Special Pathologist Cook County Insane Asylum


CHICAGO MEDICAL

JOURNAL & EXAMINER.

VOL. XLVIII.—FEBRUARY, 1884.—NO. 2.

### Original Communications.

ARTICLE I.

### Clinical and Pathological Reports of Cases of Insanity.

By S. V. Clevenger, M.D., Special Pathologist Cook County Insane Asylum.

Three hundred and thirty-seven males, and three hundred and ninety-seven females, together, seven hundred and thirty-four, insane persons have been under observation at this hospital since May, 1883. Of this number five hundred and fifty-four are present at the close of the calendar year, December 31, 1883.

During these past seven months the histories of nearly three-fourths of the patients have been collected in large volumes, and every available source of information ransacked, main reliance being placed upon narrations of relatives and physicians who previously attended the cases; but it sometimes happens that either no friends of the lunatic call, or that nothing can be elicited from them through their stupidity or unwillingness to impart information from various motives. This, fortunately, is not usual. Useful clues are afforded by the stenographer's reports of the trials on file at the county agent's office. About half the patients themselves are able to give, with more or less reliability, an account of their past lives, and from the remainder, except in cases of dementia, occasionally something can be learned also. The attendants soon learn the peculiarities of those intrusted to their care, and report to the physicians on their rounds, or at once if necessary.

The strictly medical duties of the physicians are multifarious enough for a corps of specialists. Surgery, ophthalmology, otology, dermatology, gynæcology, and rarely obstetric aid are called into requisition.

Where an autopsy is justified by the history of the case and *intra viam* observation, all departures from the normal are carefully noted, whether occurring in the brain or trunk viscera. The former is placed in a solution of kali bichromas, often changed, and tben transferred to methyl, and finally ethyl alcohol. This prevents the precipitation of the phosphorized and nitrogenous constituents of the brain, and ensures an equable hardening. The ventricles must first be injected and the fissures separated to permit access of the fluids. By the second or third month, if the pia can be separated with the forceps, sections may be cut. In the laboratory, a modification of Gudden's water microtome is used, which I described and exhibited at the last meeting of the American Microscopical Society. Sections of one-thousandth of an inch are floated off and boiled in a water bath with a carmine solution for about two hours, then washed and treated with a little glacial acetic acid, washed in filtered water and transferred to alcohol, and finally to clove oil till clear. Mountings are made in Canada balsam or glycerine jelly, and the slides forwarded to physicians interested in the study of the pathology of insanity.

From time to time cases illustrative of the various phases of mental disease and their treatment will be published in this journal, such matters as are of interest only to specialists being produced in the *Journal of Neurology and Psychiatry*, *Alienist and Neurologist*, *and Journal of Nervous and Mental Disease.*

Female case 275. Delirium grave, published in the *Journal of Neurology and Psychiatry*, August, 1883, was republished in this journal November, 1883.

Female case 238. Paretic dementia, published in the *Alienist and Neurologist*, January, 1884.

Cases of insanity in children from the ages of six to sixteen, published in the *Journal of Neurology and Psychiatry*, November, 1883.

Male case 227. Melancholia from lead poisoning. John H., aged 51, Swede, single, admitted June 7, 1883, presented the appearance of apathy and mutism observable in ordinary melancholia, with the addition of epileptiform convulsions. A friend visited him, and from him it was learned that the patient had been working in a lead factory on State street for three years. An examination revealed the characteristic blue discoloration of the gums and other symptoms of lead poisoning. He was given iodide of potassium, tonics and bathed frequently. He steadily improved, and was discharged fully recovered August 31, 1883.

Sanitary boards would do well to examine into the conduct of lead factories, and insist upon proper measures being adopted to protect workmen against plumbic toxæmia. Had the cause of this attack been unascertained, the patient would have died insane.

Male case 250. Monomania. R. D., admitted July 12, 1883; age, 32; Scotch; book-keeper; single. We were somewhat surprised to see this gentlemanly-looking fellow standing in the main hall the day of his admission, among others committed to the asylum. He spoke so intelligently, with a slightly dejected air, we concluded to make him comfortable first, and next day get his story from him. Dr. Thuemmler informed me that he had obtained the first volume of it next morning, and we should go together to hear the remainder. I told the patient that Dr. T. had detailed all that had been told him, and to proceed where he left off. The thousand and one nights' tales of Scheherazade were nothing to it in length, nor could Mark Twain's ram story equal it in digression. The substance of it was that he was an honest, good fellow, who by too much religious zeal, laboring hard to become a sermonizer in the Methodist church, ignoring completely physiological demands, came to have a mature bee in his bonnet. He sent missives of love to a lady in the congregation, who rejected his suit. This he took greatly to heart. A certain Rev. J. T. Inman, Station D, New York, insisted upon spending several thousand dollars in advertising how to cure the errors of youth, merely to annoy our patient. Wherever he went that horrible advertisement appeared, and finally stared him in the face from his native village paper in Scotland.

He went back to Montreal, and several clergyman began throwing out insinuations against his purity, in their sermons. He came to Chicago, and between Inman's advertisement and the Methodist clergymen all preaching at him, he became disgusted and went to London. There he committed the horrible crime of having an involuntary seminal ejaculation, upon seeing two good looking young ladies in the same stage with him. He had the location near Hyde Park entrance, the number of the omnibus, and the minute and hour it occurred, as exact as the recording angel could have had it. He shipped immediately for the Indian archipelago with a bluff old sea captain, who constantly insinuated that our patient was not as pure as he might be. Thence he went to Cape Town, South Africa, where he was engaged by a book-dealer, who fancied him greatly, and with whom he would have done well had it not been for the appearance of that cursed Inman advertisement in the colony paper. He even saw it in Boerish Dutch. He fled back to America, and even began to dislike going to church (another high crime which he bewailed) because of the still more direct preaching at him he encountered everywhere. Even the published sermons alluded to him. On a lake vessel the purser lost some money from his office. Forthwith our patient knew *he* was suspected of having stolen it, and now the police became the objects of his hatred. They passed his room window singly and by twos all day and night, and from what scraps of conversation he could overhear, he knew they talked only of him and his probable theft. He came to Chicago, and noticed how the police followed him and eyed him askance, and finally one policeman had the impertinence to get upon the same street car he did. Outraged nature could bear no more. He knocked the city guardian into the gutter, and was promptly arrested. He told his tale of persecution, and was brought here.

In July, 1883, a slight pneumonia attack laid him up for awhile, and upon his recovery I took him into my office, where he worked faithfully on statistics and medical records till his discharge, August 24, 1883. By regulating his habits, and with the use of tonics, he improved rapidly. I selected books for him, and by means of long conversations tried to improve his mental background until he acknowledged his delusion, and as I bade him good-bye on the train he told me all he feared was a return of the delusions in some new form. This was a good indication of mentality coming to his rescue, and I told him his only hope was self-education, a cultivation of his reasoning powers, and holding in check emotionalism of all kinds. He wrote to me from Montreal that he was about to embark for Scotland, his home, and that he was quite well. The September following he sent me an article he had written for a Scotch newspaper.

He had been treated some years before by Dr. Yellowlees, the famous Scotch alienist, for melancholia, a condition his evident monomania simulated in the last attack through the depression his vagaries engendered. It must not be forgotten that a monomaniac may, by reason of his delusions, be depressed, and that a melancholy person who *reasons* in a perverted way upon the cause of his delusions is a monomaniac, and not a melancholiac. A term which would express logical perversion would best suit this malady, as a monomaniac may have a dozen delusions, though he usually has one prominent at a time about which he is always ready to argue. This case is instructive in the mentality possessed by the patient, which could be appealed to for his future good. He read works on his disorder intelligently, and recognized the fact that his mind was disordered, and even called my attention to descriptions fitting his case, There are instances of patients hiding their delusions for the purpose of gaining freedom, but he did nothing of the kind; his frankness and dread lest the errors should return were good guarantees of his recovery. Torpidity of the bowels and sluggish circulation from which he suffered upon his arrival were corrected by strychnia.

Male Case 188. Mania. Joseph C., admitted March 28, 1883; aged 24; Bohemian; shoemaker; married. The *furor maniacorum* in this case was intense, and would come on without warning, When sitting or standing in the grounds with other patients an outbreak of maniacal rage would seize him, and often six attendants were necessary to control him. Was crafty and agile. Escaped in June, 1883, by bolting through door; ran up ladder to roof and danced over the slates and chimneys at its dizzy height with a strait-jacket on. He was baited with a plug of tobacco; as he attempted to take it with his teeth his feet were pinioned. Dr. Theummler has found great benefit to accrue from the use of Squibbs' fl. ext. conium in this case, ten minim doses *ter in die*. When his dose was missed he became violent again. August, 1883, apparently recovered, but as once before he had a remission of two weeks he is detained under observation a few weeks longer. Is now quiet, industrious and rational. He blames his insanity upon something he drank in a restaurant, but doubtless with little reason. Discharged appar ently fully recovered September 24, 1883.

Male Case 56. Mania from traumatism. Patrick K., admitted Nov. 1, 1878, aged 44: Irish; policeman. Has grandiose delusions and others which partake of his former police duties. For instance, in the winter of 1880 I sat beside him to examine him, when he addressed himself and suspicions to Dr. Spray, the superintendent. He insisted that the superintendent had some plot concerning us. He was shot in the neck by a burglar at the Halsted-street bridge October 30, 1874. He had been on the force for ten years. Dr. H. M. Bannister published this case in the *Journal of Nervous and Mental Disease*, p. 434, July, 1879, as insanity resulting from a wound of the cervical sympathetic. The scar is just anterior to left sterno-cleido-mastoid muscle two inches above its insertion. He is extremely suspicious and abusive, but otherwise harmless. There are no traces now of the flushing which Dr. B. noticed on the left side of the face. Incurable.

Male case 218. Paretic Dementia. P. M., admitted May 22, 1883, age 40; Norway; physician; single. A classmate of the patient stated that he had decided paretic symptoms two years ago; hesitancy of speech, grand delusions and amnesic periods, and that he had been arrested and fined repeatedly for publicly urinating in the streets. He attacked a saloon keeper fiercely and was brought here. He several times micturated against wall of ward and denied all knowledge of such acts when accused of them. Having great strength he broke a corridor door through in an attempt at escape, but calmed down when reasoned with and convinced that he had been tried by jury and found guilty of being insane (so we may construe the operation of our State laws). His delusions of grandeur were intellectual; says he was four years assistant physician in the Christiania asylum, which is quite probable; fancies himself a great gynæcologist; occasionally imagines himself very wealthy. He stood well in medical circles, and really was a good surgeon, but while here showed little interest in anything beyond his meals and the trivialities of his surroundings. Occasional attacks of furor were followed by epileptiform convulsions. An aunt on mother's side had an indefinite mental complaint showing probable heredity predisposition. When he spoke his face would suffuse with blood, this capillary flushing patient stated was inherited. He succumbed to an attack of typhoid pneumonia September 10, 1883. Autopsy ten hours after death: Scalp thick, and more than ordinary quantity of blood oozed, or rather, ran from incisions. Skull very thick and dense, opened with difficulty. Left cerebrum præ-frontal region large extravasations, imparting a scarlet flush, extended from base to first frontal fissure and to operculum nearly to sulcus of Rolando. Leucocytic exudation general, adhesions of dura to brain in region of superior longitudinal sinus, summit of dura full of hæmatoidin crystals. One pacchionian body on left side just anterior to sulcus Rolando. Occipital region capillary stasis great. but relatively less than in frontal and parietal region. Small arterial rupture near pons, base of temporal region. Right cerebrum venous stasis on summit more marked than on left, a general flush about one inch anterior to præcentral sulcus extending backward, not involving the temporal lobe more than occipital. Isthmus extremely large. Pons bowed out unusually (normal for this brain). Total brain weight 57 ounces.

Sections microscopically examined showed the usual evidences of paretic dementia, such as thromboses, kinked, distorted blood-vessels with perivascular channels (now established as pathological not physiological). The vascular fluctuations observed in his face during life were similarly occurring in his brain, with the addition of ruptures and tortuosities due to feebler support of contiguous tissue. Intemperance was an element in his case, but whether causative of or consequent upon his insanity, certainly it was an aggravating factor.

Male Case 247. Epileptic Insanity. F. S., admitted July 5, 1883, aged 6 years—youngest case on record in this asylum. American. Epileptic since one year old. Maniacal; restless; fights and cries when opposed or taken hold of. Does not speak. Habits filthy, mischievous. Notices very little, but attention attracted by noises. Health and physique otherwise good. Mother appears indifferently balanced. Under kali bromidum quieted down rapidly, and was taken home 3 days after admission, improved but seeming dazed. The child had evidently had no proper treatment previously.

Male Case 260. Epileptic Insanity. A. M., admitted July 26, 1883. American. Aged 24 years. Hostler. Single. Epileptic 6 years. Often one paroxysm in 3 or 4 weeks, and, if excited, 2 or 3 daily. Body spotted with tricophytosis corporis. Mother, brother, and sister scrofulous. Amnesic. Aura starts with whirring noise in head. Hallucinations of hearing. Repeats the same sentence over many times, but otherwise apparently sane. Was industrious and peaceable till December 14, 1883, when, after several severe epileptic attacks, he grew violent and struck an attendant, exhibited extreme restlessness, turning round often, lapsed into stupidity, alternately with fits, 2 or 3 daily; but by the last of December is improving.

Male Case 214. Katatonia. F. S., admitted September 25, 1879. Age 39. German. Laborer. Married. Apparently demented on arrival. Emaciated, with muscular rigidity. No hereditary history of mental disease. Father died of phthisis pulm. Mother living and aged. Up to June 25, 1879, had been at work, but developed delusions of persecution—thought his associates wanted to kill him—suddenly refused to speak or eat; saliva oozed from his mouth. Pulse 120. Has to be fed. October 10, spoke rationally. Says he remembers everything that transpired during his demented state; that he feels well and happy now, but that he had painful delusions and heard a voice telling him he must keep silent for several days. October 22, became suddenly cataleptic, stupid, and nearly in same condition as when admitted. Bowels atonic, enemata necessary. October 28, improving gradually until rational again by November 25. Mentioned the imperative voice again, as having controlled his actions, warning him against moving a single joint. The condition is not catalepsy, but a cataleptoidal one which could be overcome entirely were volition not dominated by hallucinations. January 15, 1880, maniacal furor great. January 24, says he is God. February 8, still violent. February 18, improved gradually till taken home, July 11, 1880. [The foregoing observations were made by Dr. A. W. Hagenbach, the assistant physician.] Re-admitted January 13, 1881, since when the cyclical character of his attacks were pretty much as above—melancholic, cataleptoidal conditions and mania, December 31, 1883, passing into the cataleptoidal state, preceded by quiet for 3 months. His mind is weaker than formerly, but his bodily condition is better, having grown stouter.

